# GPX4-Regulated Ferroptosis Mediates S100-Induced Experimental Autoimmune Hepatitis Associated with the Nrf2/HO-1 Signaling Pathway

**DOI:** 10.1155/2021/6551069

**Published:** 2021-12-20

**Authors:** Lujian Zhu, Dazhi Chen, Yin Zhu, Tongtong Pan, Dingchao Xia, Tingchen Cai, Hongwei Lin, Jing Lin, Xiaozhi Jin, Faling Wu, Sijie Yu, Kailu Zhu, Lanman Xu, Yongping Chen

**Affiliations:** ^1^Department of Infectious Diseases, The First Affiliated Hospital of Wenzhou Medical University, Zhejiang Provincial Key Laboratory for Accurate Diagnosis and Treatment of Chronic Liver Diseases, Wenzhou Key Laboratory of Hepatology, Hepatology Institute of Wenzhou Medical University, Wenzhou, China; ^2^Department of Gastroenterology, Peking University First Hospital, Beijing, China; ^3^Department of Infectious Diseases and Liver Diseases, Ningbo Medical Centre Lihuili Hospital, Affiliated Lihuili Hospital of Ningbo University, Ningbo Institute of Innovation for Combined Medicine and Engineering, Ningbo, China

## Abstract

Autoimmune hepatitis (AIH) is an inflammatory autoimmune disease of the liver. Oxidative stress triggered by reactive oxygen radicals is a common pathophysiological basis for the pathogenesis of many liver diseases, and ferroptosis is associated with the toxic accumulation of reactive oxygen species. The signaling transduction pathways responsible for iron processing and lipid-peroxidation mechanisms are believed to drive ferroptosis. However, the specific mechanisms regulating ferroptosis remain unclear. The aims of this investigation were to identify the possible effector functions of ferroptosis, based on glutathione peroxidase 4 (GPX4) regulation in an S100-induced autoimmune hepatitis mouse model and hepatocyte injury models. The S100 liver antigen-induced AIH mouse model was used to detect ferroptotic biomarkers using western blotting. Upregulated levels of cyclooxygenase2 (COX2) and Acyl-Coenzyme A synthase long-chain family member 4 (ACSL4) were observed in the S100-induced AIH model group, while levels of GPX4 and ferritin heavy chain 1 (FTH1) were downregulated (*P* < 0.05). The expression profiles of COX2, ACSL4, GPX4, and FTH1 were restored following the administration of ferrostatin-1. In addition, Nrf2 and HO-1 levels in the S100-induced AIH model mice after treatment with ferrostatin-1 were downregulated compared to the nonferrostatin-1-treated S100-induced AIH model mice (*P* < 0.05). Moreover, COX2 and ACSL4 levels were significantly upregulated, with significant FTH1 downregulation, in the AIH model mice when liver-specific GPX4 was silenced using AAV8 constructs. These data indicate that inhibition of ferroptosis significantly ameliorated the influence of AIH on the Nuclear factor E2-related factor 2 (Nrf2)/Heme oxygenase-1 (HO-1) signaling pathway, and that ferroptosis may act as an initiator or intermediate mediator leading to AIH.

## 1. Introduction

Autoimmune hepatitis (AIH) is characterized by inflammation, the presence of autoantibodies, hypergammaglobulinemia, and interface hepatitis [1]. It is more common in women and appears to be increasing in increasing prevalence. Chronic liver disease is a serious threat to health and thus has economic consequences [2]. Although the pathogenetic mechanism of AIH is not clear, the mouse AIH model induced by the liver-specific antigen S100 is widely used to study AIH [3].

Typically, mice which are injected intraperitoneally with equal amounts of liver S100 antigen emulsified and mixed with complete Freund's adjuvant on day-0 and day-7 show significant liver inflammation and elevated autoimmune IgG. Although previous studies have implicated genetic susceptibility, molecular mimicry, as well as external factors in AIH pathogenesis [4], the precise pathogenetic mechanism remains unclear. Therefore, an in-depth study of the underlying mechanisms responsible for AIH can assist the development of novel therapies for AIH.

Ferroptosis is defined as iron-dependent cell death mediated by phospholipid peroxidation. Ferroptosis results from dysregulated iron metabolism involving the iron-storage protein ferritin, composed of a ferritin light chain (FTL) and a ferritin heavy chain 1 (FTH1) [5]. Since FTH possesses iron oxidase activity, it converts ferrous iron (Fe^2+^) to the oxidized ferric (Fe^3+^) form, allowing iron binding to the ferritin outer layer and thus lowering the concentration of free iron in the cell [6]. High levels of free iron are associated with lipid peroxidation. There are several mechanisms involved in the prevention of lipid peroxidation including the action of glutathione peroxidase 4 (GPX4) that scavenges phospholipid peroxides as a protective measure against ferroptosis [7]. Abnormal iron metabolism and lipid peroxidation are believed to play pivotal parts in ferroptosis. In addition, lipid peroxidation is influenced by a spectrum of different lipids and enzymes [8]. Even though lipid peroxidation acts as a key process driving ferroptosis, the regulators of lipid metabolism in ferroptosis remain unclear [9]. Acyl-Coenzyme A synthetase long-chain family member 4 (ACSL4) is essential for apoptosis in iron chain cancer cells. ACSL4 expression has been shown to be significantly reduced in ferroptosis-resistant cells [10]. Thus, ACSL4 participates in the catalysis of polyunsaturated fatty acid oxidation. The enzyme lysophosphatidylcholine acyltransferase 3 (LPCA T3) mediates lipotoxicity in ferroptosis [10–12]. Furthermore, several intracellular signaling pathways play pivotal parts in ferroptosis. For example, investigations have implicated ferroptosis in pathological conditions in a variety of organs, including the brain, kidney, liver, and heart [13]. Ferroptosis is seen in the accumulation of iron and reactive oxygen species (ROS) and inhibits the activities of the XC- and GPX4 systems by reducing cysteine uptake and consuming glutathione [14]. The harmful actions of lipid peroxidation during the actual ferroptotic process can be inhibited through lipophilic-free radical traps (such as vitamin E, ferrostatin-1, and liproxstatin-1). However, the influence of ferroptosis on AIH remains unclear. Nuclear factor erythroid-related factor 2 (Nrf2) is a transcription factor that maintains stability and homeostasis in stressed intracellular environments, and, although it appears to be involved, its underlying mechanism of action is unknown [15]. Increasing evidence has implicated Nrf2 in ferroptosis pathogenesis [16]. In our present study, heme oxygenase-1 expression was found to be significantly increased in the livers of S100-induced AIH mice, while other studies have suggested that excessive Nrf2/HO-1 stimulation can lead to ferroptosis by disrupting the balance of ferric ions [17, 18]. Here, we hypothesized that ferroptosis plays a pivotal role in Nrf2/HO-1 signaling in S100-induced AIH. Therefore, in the current investigation, ferroptosis and its mechanism were evaluated in the AIH mouse model to determine its possible pathogenic role in AIH and to generate novel therapies for patients with AIH.

## 2. Materials and Methods

### 2.1. Animal Models

Male C57BL/6 WT mice weighing 23-25 g were purchased from Hangzhou Ziyuan Experimental Animal Technology Co, Ltd. (China). The study and animal handling were approved by the Animal Policy and Welfare Committee of Wenzhou Medical University (approval document no. wydw2020-0861). The care of the mice was in accordance with NIH guidelines (Guide for the Care and Use of Laboratory Animals). The mice were housed in a pathogen-free environment with a 12 : 12 h light-dark cycled and fed on standard rodent chow. All mice were allowed to acclimatize to their new environment for a minimum of 14 days prior to the commencement of the AIH modeling. Ten experimental mice were randomly selected to be given an intraperitoneal injection of S100 injection, thus, comprising the mouse AIH model group.

### 2.2. Preparation of Mouse Liver Tissue

**Figure 1:**
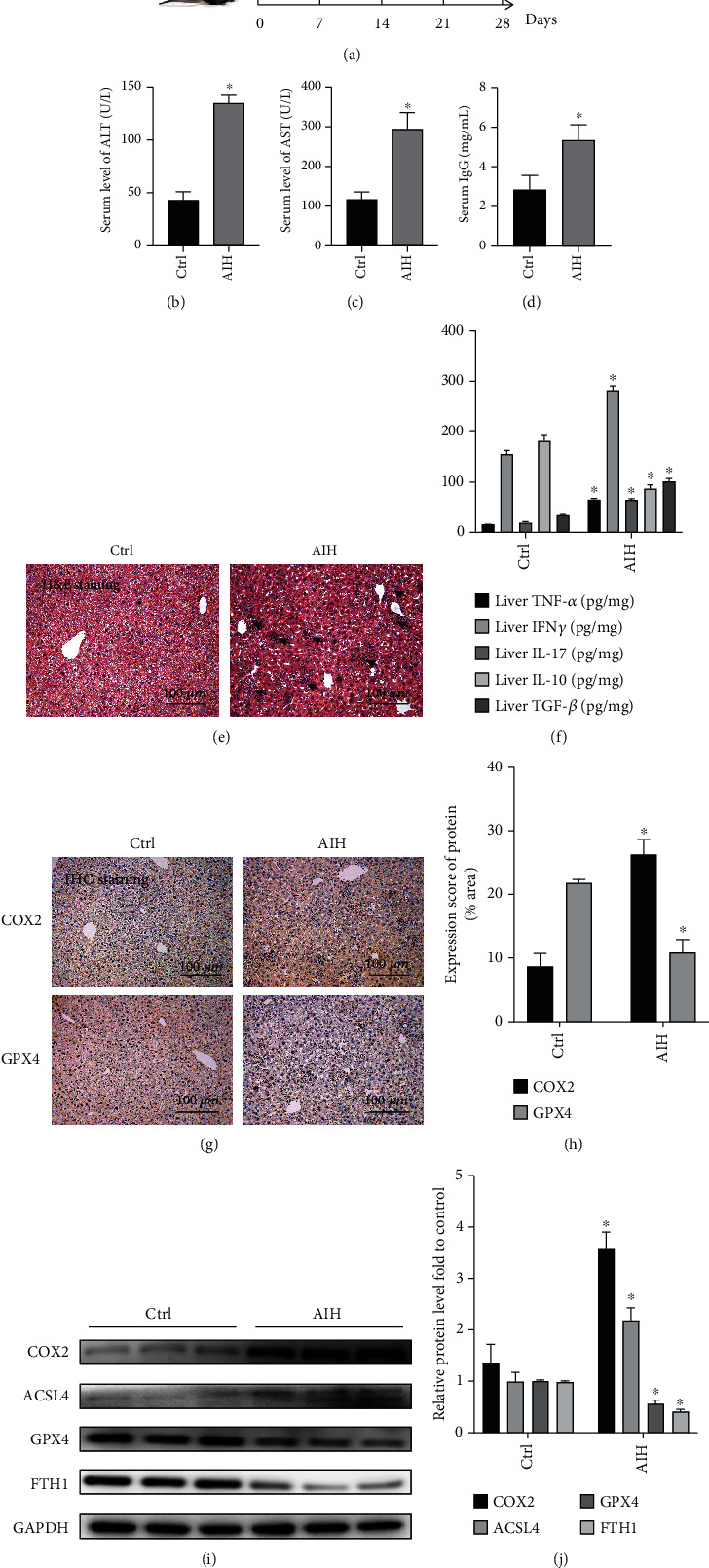
Ferroptosis plays an important role in S100-induced autoimmune hepatitis. (a) Experimental protocol for the modeling of S100-induced AIH model mice. (b)–(d) The serum ALT, AST, and IgG expression levels in the control and AIH groups. (e) Representative H&E staining of liver tissue sections. The black arrow indicates lymphocytic infiltration (original magnification 20×). (f) TNF-*α*, IFN*γ*, IL-17, IL-10, and TGF-*β* levels in liver. (g) IHC staining of COX2 and GPX4 in liver sections (original magnification 20×). (h) Semiquantitative IHC results. (i)–(j) Western blotting showing protein expression of COX2, ACSL4, GPX4, and FTH1 in the pre-experimental control and AIH groups. GAPDH was used as a loading control; ^∗^*P* < 0.05, compared with the control group.

To prepare the liver-specific antigen S100, three mice were randomly selected and sacrificed under pentobarbital sodium anesthesia. The livers were perfused with phosphate-buffered saline (PBS) and removed as previously described and used to prepare fresh S100 antigen [3]. In brief, the liver was homogenized in precooled PBS, followed by a 60-minute centrifugation at 150 g. The supernatant was further centrifuged for 60 minutes in an ultracentrifuge (100 000 g) [3]. The S100-containing supernatant was concentrated to 5 mL through an Amicon® Ultra-15 ultrafiltration system (Millipore, USA), followed by separation on an AKTA Pure (GE Healthcare, USA) 90 cm CL-6B Sepharose® column (Pharmacia, USA). Three protein peaks were eluted from the column, with peak 2 being the harmful component and peaks 1 and 3 the required and nontoxic components, respectively, of the hepatic antigen. The protein fraction from the first peak was used at a concentration of 0.5-2.0 g/L. For the establishment of the AIH model, the hepatic S100 antigen was emulsified with an equal volume of Freund's complete adjuvant (Solarbio, China) and was used to immunize 10 mice from the same batch intraperitoneally on day-0 and day-7 (Figure 1(a)). During the modeling, two of the mice in the AIH model group died. Four weeks after the injection, the mice were sacrificed under pentobarbital sodium anesthesia. Blood samples were collected and centrifuged at 3000 rpm at 4°C for 15 min to obtain the serum. Liver samples were either fixed in 4% paraformaldehyde for immunohistochemical and histological examination or frozen at -80°C.

### 2.3. Ferrostatin-1 Treatment of Mice

Ten control mice were fed with conventional rodent chow and water throughout the modeling period. Experiments were conducted to further demonstrate whether ferroptosis occurs in S100-induced AIH and whether intervention with ferrostatin-1 intervention is able to ameliorate it. The mice in the experimental group received intraperitoneally injected ferrostatin-1 (1 mg/kg body weight in 5% DMSO) [19].

**Figure 2:**
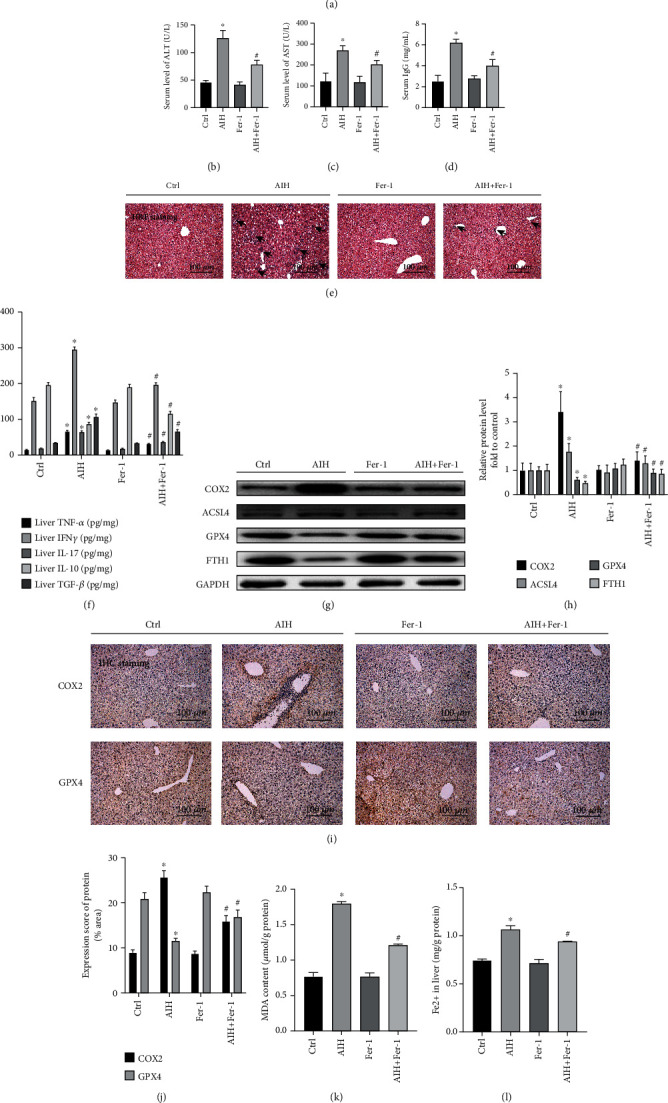
Ferrostatin-1, a ferroptosis inhibitor, significantly improves S100-induced autoimmune hepatitis. (a) Experimental protocol for Ferrostatin-1 treatment of S100-induced AIH model mice. (b)–(d) ALT, AST, and IgG levels in the control group, AIH group, Ferrostatin-1-treated group, and AIH + Ferrostatin-1 group are shown. (e) Representative H&E staining of liver tissue sections. The black arrow indicates the lymphocytic infiltration (original magnification 20×). (f)TNF-*α*, IFN*γ*, IL-17, IL-10, and TGF-*β* levels in liver; (g) and (h) Western blot showing protein expression of COX2, ACSL4, GPX4, and FTH1 in the control, AIH, Ferrostatin-1, and AIH + Ferrostatin-1 groups; GAPDH was used as a loading control. (i) IHC images of COX2 and GPX4 in liver sections (original magnification 20×). (j) Semiquantitative IHC results. (k) Detection of lipid peroxidation by measuring malondialdehyde (MDA) levels. (l) Fe^2+^ levels in liver; ^∗^*P* < 0.05, compared with control group; ^#^*P* < 0.05, compared with AIH group.

The following experimental groups were used: (i) normal control group, (ii) S100-induced AIH model group, (iii) normal mice + Ferrostatin-1 intervention group, and (iv) S100-induced AIH + Ferrostatin-1 treatment group. A total of 40 experimental mice were used with 10 mice in each group. The AIH model was established as described above. Ferrostatin-1 was injected intraperitoneally as shown in Figure 2(a), using the same dose and procedure for both normal and AIH mice. After completion of the Ferrostatin-1 intervention, the four groups of mice were sacrificed under pentobarbital anesthesia at the same time, and blood samples and liver tissues were collected for further experimental analysis.

### 2.4. AAV8-m-GPX4 Treatment of Mice

**Figure 3:**
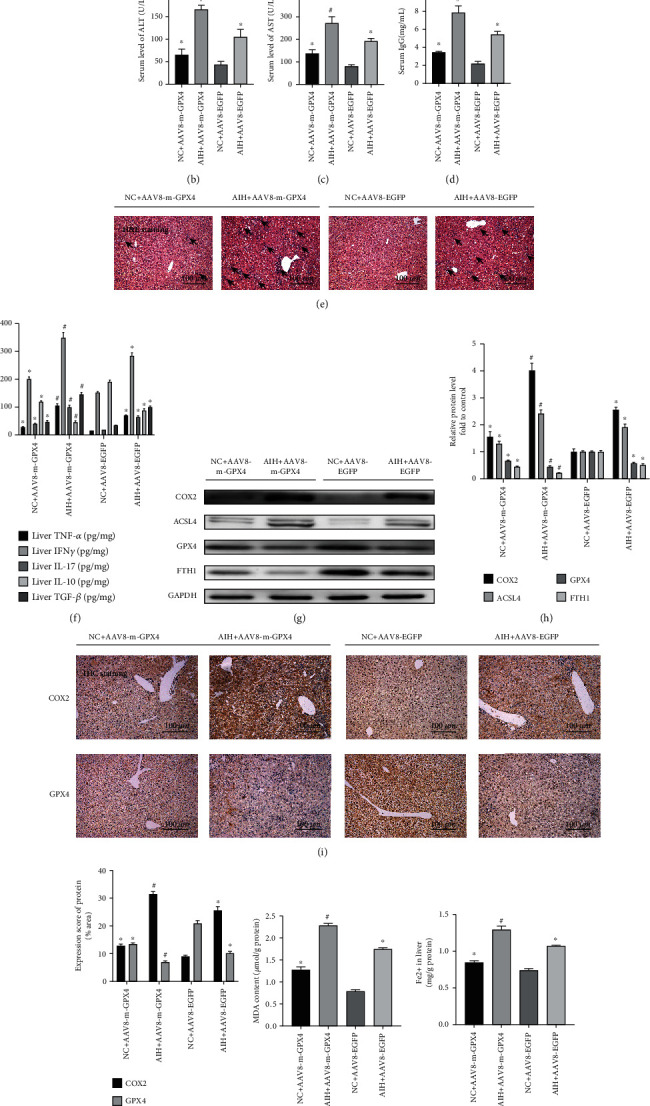
Exacerbation of S100-induced autoimmune hepatitis after GPX4 knockdown. (a) Experimental protocol for the transfection of AAV8-m-GPX4 and the establishment of the mouse AIH model; (b)–(d) ALT, AST, and IgG levels in the NC + AAV8-m-GPX4, AIH + AAV8-m-GPX4, NC + AAV8-EGFP, and AIH + AAV8-EGFP groups. (e) Representative H&E staining of liver tissue sections. The black arrow indicates lymphocytic infiltration (original magnification 20×); (f) TNF-*α*, IFN*γ*, IL-17, IL-10, and TGF-*β* levels in liver; (g) and (h) Western blot showing protein expression of COX2, ACSL4, GPX4, and FTH1 in the NC + AAV8-m-GPX4, AIH + AAV8-m-GPX4, NC + AAV8-EGFP, and AIH + AAV8-EGFP groups; GAPDH was used as a loading control. (i) IHC images of COX2 and GPX4 in liver sections (original magnification 20×). (j) Semiquantitative IHC results. (k) Detection of lipid peroxidation by measuring malondialdehyde (MDA) levels. (l) Fe^2+^ levels in liver; ^∗^*P* < 0.05, compared with the NC + AAV8-EGFP group; ^#^*P* < 0.05, compared with the AIH + AAV8-EGFP group.

AAV8-m-GPX4 was used to silence GPX4 gene expression to investigate whether S100-induced ferroptosis in AIH is associated with GPX4 expression. The experimental groups were as follows: (i) normal mice + AAV8-m-GPX4 group, (ii) AIH + AAV8-m-GPX4 group, (iii) normal mice + AAV8-negative control group, and (iv) AIH + AAV8-negative control group. A total of 40 mice were used with 10 mice in each group. The timing of the AAV injection is shown in Figure 3(a). Four weeks after the first intraperitoneal injection of S100, the four groups of mice were sacrificed, and blood and hepatic tissue samples were extracted for further experimental analysis.

### 2.5. H&E Staining

The liver tissue was fixed with 4% paraformaldehyde, embedded in paraffin, and 5 *μ*m sections cut. The sections were stained with hematoxylin and eosin (H&E). The lymphocytic infiltration level, inflammatory necrosis, and destruction of liver structures were observed under light microscopy (Olympus, Japan).

### 2.6. Enzyme-Linked Immunosorbent Assay (ELISA)

The concentrations of the inflammatory cytokines (TNF-*α*, IFN*γ*, and IL-17), the fibrotic cytokine TGF-*β*, and the anti-inflammatory cytokine IL-10 that play important roles in AIH progression were measured in liver tissue lysates. The following mouse ELISA kits were used, all from Multisciences (Lianke) Biotech (Hangzhou, China): TNF-*α* (EK282/3-48), IFN*γ* (EK280/3-48), IL-17 (EK217/2-48), IL-10 (EK210/4-48), and TGF-*β* (EK981-48), according to the manufacturer's instructions. Although different kits have different methodologies for the assay, the main experimental steps are similar. Hereby, we use the measurement of TNF-*α* concentration as an example of ELISA method. In brief, TNF-*α* standard, blank control, and sample under test (100 *μ*l/well) were added to a 96-well plate. Then, add 50 *μ*l dilution of antibody to each well (1 : 100 dilution). Seal the plates with a plate sealing membrane and incubate at room temperature for 90 minutes. After washing off the unbound biotinylated antibody, add 100 *μ*l labeled Streptavidin-HRP to each well (1 : 100 dilution) and incubate at room temperature for 30 minutes. After washing, add 100 *μ*l substrate TMB to each well and incubate at room temperature for 15 minutes, avoiding light. The reaction was terminated by the termination reagent and the optical density (OD) value was measured by an enzyme marker at the wavelength of a 450 nm. The concentration of cellular inflammatory factors was computed using the standard curve regression equation of standard absorbance values.

### 2.7. Western Blot Analysis

The liver tissue was homogenized in lysis buffer. After centrifugation, the protein concentration of the lysate was measured using BCA Protein Assay Kit (Beyotime, P0012, China), in line with the manufacturer's protocols. Equivalent amounts (50 *μ*g) of protein were separated on SDS-PAGE and transferred to PVDF membranes (Millipore). The membranes were blocked with 5% skimmed milk in Tris-buffered saline with 0.1% Tween 20 (TBST) and incubated with antibodies against COX2 (1 : 1000, 12375-1-AP, Proteintech), ACSL4 (1 : 1000, A16848, ABclonal), GPX4 (1 : 1000, BM5231, Boster), FTH1 (1 : 1000, A19544, ABclonal), Nrf2 (1 : 1000, 16396-1-AP, Proteintech), HO-1 (1 : 1000, 10701-1-AP, Proteintech), or GAPDH (1 : 10 000, 60004-1-AP, Proteintech) overnight at 4°C. After washing, the membranes were incubated with horseradish peroxidase- (HRP-) conjugated secondary antibodies against rabbit or mouse IgG (1 : 5000, LF101 and LF102, respectively, Epizyme) for 1 hour at room temperature. A Bio-Rad immunoblot analysis detection system (Bio-Rad, Hercules, CA, USA) was used for visualization. Protein band densities were assessed by ImageJ analysis software, and the relative densities against the loading control (GAPDH) were calculated.

### 2.8. Real-Time Quantitative Polymerase Chain Reaction (RT-qPCR)

Total RNA was collected from homogenized liver tissue with TRIzol® reagent, and 1 g total RNA was reverse-transcribed into cDNA using the PrimeScript RT Master Mix® (Perfect Real Time) kit (RR036A, Takara, Japan). The quantitative polymerase chain reaction was performed in a 10 *μ*L reaction mixture containing specific primers and TBGreen Premix Ex Taq II. Amplification was performed in a real-time fluorescent quantitative PCR system (AB 7500). The primers employed consisted of the following:

ACTB (5′-CCTCACTGTCCACCTTCC-3′, 5′-GGGTGTAAAACGCAGCTC-3′),

Nrf2 (5′-TCTTCACTGCCCCTCATC-3′, 5′-CTCCTGCCAAACTTGCTC-3′),

HO-1 (5′-ACAGCCCCCCACCAAGTTC-3′, 5′-GGCGGTCTTAGCCTCTTC-3′).

Datasets were assessed using the 7500 real-time PCR system software. Actin levels were used for normalization. Three technical replicates of each RT-qPCR analysis were performed.

### 2.9. Immunohistochemistry (IHC)

Immunohistochemistry was performed on paraffin-embedded mouse liver sections. The semiquantitative IHC datasets were based on the mean of three mice per group. Three sections from each mouse were assessed, with imaging collected by optical microscopy (Olympus, Japan). The staining intensity was analyzed using ImageJ software, and three microscopy fields were chosen at random to calculate the integrated optical density (IOD) for the target protein.

### 2.10. Serum Transaminases and IgG Analyses

Serum was collected from blood samples by centrifugation at 250 g for 10 minutes. Serumalanineaminotransferase (ALT) and aspartate aminotransferase (AST) concentrations were analyzed using a fully automated biochemical analyzer, following the manufacturer's protocols (Abbott Laboratories, Chicago, IL, USA). Serum IgG levels were determined with the Mouse ELISA Kit (EK271-48, Multisciences). All experiments were performed in accordance with the manufacturer's instructions.

### 2.11. Lipid Peroxidation Malondialdehyde (MDA) Assay

Histones were isolated using the Lipid Peroxidation Malondialdehyde Assay Kit (Beyotime, S0131, China). The malondialdehyde (MDA) concentration of each sample was measured at 532 nm using an enzyme marker (Thermo Multiskan MK3, Thermo Fisher, Waltham, MA, USA), and 490 nm was used as a control.

### 2.12. Iron Load Assay

Histones were isolated using the Iron Assay Kit protocol (ab83366, Abcam, Cambridge, UK). Liver tissue (10 mg) was washed with prechilled PBS and homogenized with 4-10 parts iron analysis buffer using a chilled Dawes homogenizer (10-15 passes). The sample was centrifuged at 16 000 g for 10 minutes. The supernatant was collected and transferred to a clean centrifuge tube. Test wells were treated with 100 *μ*L of standard dilution and sample. Iron-reducing agent (5 *μ*L) was added to each well. The kit protein standards and tissue stock solution were mixed and added to the mixtures for 30 minutes at 37°C in a constant temperature incubator. One hundred microliters of the iron probe were then added to all wells containing iron standards and test samples. The samples were mixed and incubated at 37°C for 60 minutes, away from light. The absorbance was measured on a colorimetric enzyme standard immediately afterward (OD 593 nm).

### 2.13. Cell Culture

Alpha mouse liver 12 (AML12) cells were cultured in DMEM/F-12 (1 : 1) medium containing 10% fetal bovine serum with 40 ng/mL of dexamethasone and 1% insulin-transferrin-selenium-ethanolamine (ITS-X) at 37°C in a 5% CO_2_ humidified thermostatic incubator. When the cells reached 70%-80% confluence, GPX4-specific knockdown siRNA and nonspecific control siRNA, as well as a GPX4-specific overexpression plasmid and a nonspecific control plasmid, were transfected with Lipofectamine 2000 Reagent (Thermo Fisher) strictly according to the manufacturer's instructions. After transfection, the supernatant was discarded, and incubation was continued with OPTI medium (Gibco, Thermo Fisher) for 6 hours followed by incubation for 48 hours in DMEM/F12 complete medium containing lipopolysaccharide (5 ug/ml). GPX4-specific knockdown siRNA (5′-CUGACGUAAACUACACUCATT-3′, 5′-UGAGUGUAGUUUACGUCAGTT-3′) and nonspecific control siRNA (5′-UUCUCCGAACGUGUCACGUTT-3′, 5′-ACGUGACACGUUCGGAGAATT-3′), and GPX4-specific overexpression plasmid and nonspecific control plasmid were purchased from GenePharma (Shanghai, China).

### 2.14. Immunofluorescence

AML12 cells were cultured for 48 hours in 12-well plates. After reaching approximately 80% confluence, the cells were washed with prechilled PBS and were fixed with 4% paraformaldehyde for 15 min, followed by permeabilization with 0.5% Triton X-100 in PBS for 20 min. The cells were then blocked with 5% BSA at 37°C for 1 h and incubated with the corresponding primary antibodies at 4°C overnight. The following day, the samples were incubated with Alexa Fluor 488-labeled goat anti-rabbit IgG secondary antibody (1 : 1000, 33106ES60, Teasen Biotechnology, Shanghai, China) for 1 h at room temperature in the dark, followed by incubation with DAPI for 5 minutes. Three regional fields of view were randomly selected from each fluorescent section under an ortho-fluorescent microscope (Leica, Germany), with the observer blinded to the experimental group.

### 2.15. Statistical Analysis

Statistical analyses employed GraphPad® 8.6.3 software. All studies were carried out on three separate occasions and were randomized. The *t*-test for unpaired outcomes was employed for comparative analysis. Datasets were represented as mean ± SD, with *P* < 0.05 deemed to confer statistical significance.

## 3. Results

### 3.1. Ferroptosis Plays a Pivotal Role in S100-Induced AIH

The experimental design for the establishment of the S100-induced AIH model is illustrated in Figure 1(a). The levels of ALT and AST were significantly raised in the AIH group, together with increased levels of IgG (Figures 1(b)–1(d)). Histopathological H&E staining showed that the S100-induced AIH resulted in many areas of inflammatory necrosis, increased lymphocyte infiltration, and destruction of liver structures (Figure 1(e)). ELISA analysis showed that concentrations of the inflammatory cytokines TNF-*α*, IFN*γ*, and IL-17 and the fibrotic cytokine TGF-*β* levels were significantly increased in the livers of AIH mice compared to the control group, while the anti-inflammatory cytokine IL-10 levels were significantly decreased (Figure 1(f); *P* < 0.05). Western blot and immunohistochemical assays were also used to detect the occurrence of ferroptosis in autoimmune hepatitis. The western blot results indicated upregulated expression of COX2 and ACSL4 in the AIH experimental group, while the expression of GPX4 and FTH1 was severely downregulated (Figures 1(i) and 1(j); *P* < 0.05). The immunohistochemical results showed that COX2 staining was weaker in the control mouse hepatocytes, while COX2 was significantly increased in hepatocytes in S100-induced AIH. GPX4 staining was stronger in the control mouse hepatocytes, and in the S100-induced autoimmune hepatitis model group, GPX4 expression was significantly reduced (Figures 1(g) and 1(h); *P* < 0.05). Thus, our results suggest the occurrence of ferroptosis in S100-induced autoimmune hepatitis.

### 3.2. Ferrostatin-1, a Ferroptosis Inhibitor, Significantly Improves S100-Induced Autoimmune Hepatitis

Ferrostatin-1, a ferroptosis inhibitor, was used to investigate whether ferroptosis plays a part in S100-induced AIH. The experimental protocol is illustrated in Figure 2(a). The levels of ALT, AST, and IgG were significantly raised in the AIH group compared to the blank control group (Figures 2(b)–2(d); *P* < 0.05). After Ferrostatin-1 treatment, the S100-induced autoimmune hepatitis group had significantly lower ALT, AST, and IgG levels compared with the S100-induced autoimmune hepatitis model group (*P* < 0.05). Histological H&E staining suggested that Ferrostatin-1 effectively attenuated liver damage, protected liver structures, and limited liver inflammatory lymphocyte infiltration. Ferrostatin-1 treatment attenuated inflammation in the S100-induced autoimmune hepatitis group compared to the S100-induced autoimmune hepatitis model group (Figure 2(e)). ELISA analysis showed that Ferrostatin-1 reversed the upregulated expression of the inflammatory cytokines TNF-*α*, IFN*γ*, IL-17, the fibrotic cytokine TGF-*β*, and the anti-inflammatory cytokine IL-10 in the liver of S100-induced AIH mice to some extent (Figure 2(f); *P* < 0.05). Western blotting showed that Ferrostatin-1 significantly upregulated both GPX4 and FTH1 and significantly downregulated COX2 and ACSL4 (Figures 2(g) and 2(h); *P* < 0.05). Meanwhile, the immunohistochemical results demonstrated that COX2 expression was significantly increased in hepatocytes in S100-induced AIH compared to the controls. However, COX2 expression was significantly inhibited in the S100-induced AIH group after Ferrostatin-1 treatment. In addition, Ferrostatin-1 treatment significantly increased GPX4 expression in hepatocytes (Figures 2(i) and 2(j); *P* < 0.05). In the lipid peroxidation malondialdehyde assay, Ferrostatin-1 reduced MDA levels in the S100-induced AIH group (Figure 2(k); *P* < 0.05) and also reversed to some extent the elevated levels of hepatic ferrous ions in mice after AIH induction (Figure 2(l); *P* < 0.05). Our findings further suggest that ferroptosis is associated with S100-induced AIH and that Ferrostatin-1 has a role in ameliorating S100-induced autoimmune hepatitis.

### 3.3. Ferrostatin-1 Ameliorates S100-Induced AIH via the Nrf2/HO-1 Signaling Pathway

**Figure 4:**
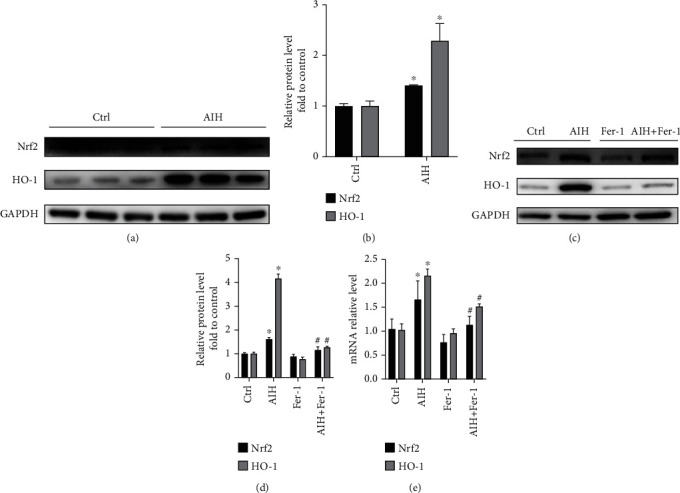
Ferrostatin-1 ameliorates S100-induced AIH via the Nrf2/HO-1 signaling pathway. (a) and (b) Western blot showing protein expression of Nrf2 and HO-1 in the pre-experimental control and AIH groups. (c) and (d) Western blot showing protein expression of Nrf2 and HO-1 in the control, AIH, Ferrostatin-1, and AIH + Ferrostatin-1 groups; GAPDH was used as a loading control. (e) RT-qPCR assay for Nrf2 and HO-1 mRNA expression in the control, AIH, Ferrostatin-1, and AIH + Ferrostatin-1 groups; ACTB was used as a loading control; ^∗^*P* < 0.05, compared with control group; ^#^*P* < 0.05, compared with AIH group.

Nrf2 plays a major part in the protection of cells from oxidative stress. Western blotting and RT-qPCR analysis revealed that both protein and mRNA expression levels of Nrf2 and HO-1 in the S100-induced AIH group were increased. However, more notably, S100-induced AIH significantly inhibited both Nrf2 and HO-1 protein and mRNA levels after Ferrostatin-1 treatment compared to the S100-induced AIH model group (Figures 4(a)–4(e); *P* < 0.05).

### 3.4. Aggravation of S100-Induced Autoimmune Hepatitis after GPX4 Knockdown

Two viruses, AAV8-m-GPX4 and AAV8-EGFP, were purchased from HANBIO [20]. The HANBIO constructs comprised a GPX4 knockdown sequence in an adeno-associated virus (AAV). Forty male C57BL/6 mice were randomly divided into four groups, (i) NC + AAV8-m-GPX4, (ii) AIH + AAV8-m-GPX4, (iii) NC + AAV8-EGFP, and (iv) AIH + AAV8-EGFP, with 10 mice in each group. Each mouse was injected with 1 × 10^12^ copies of the virus. The experimental protocol for transfection of AVVs and establishment of S100-induced autoimmune hepatitis model in mice is shown in Figure 3(a).

AAV8-m-GPX4 was used to interfere with the expression of GPX4 in knockdown normal controls and in the S100-induced autoimmune hepatitis model to verify the role of the GPX4 protein in S100-induced AIH and ferroptosis development. The expression of ALT, AST, and IgG in the AIH + AAV8-m-GPX4 group was significantly increased in comparison to the NC + AAV8-m-GPX4 group (Figures 3(b)–3(d); *P* < 0.05). However, notably, the NC + AAV8-m-GPX4 group showed slightly higher expression of ALT, AST, and IgG than the NC + AAV8-EGFP group (*P* < 0.05). In addition, the results of the H&E pathological staining suggested that liver damage, liver structural destruction, and liver lymphocyte infiltration were more severe in the AIH + AAV8-m-GPX4 group compared to the AIH + AAV8-EGFP group. Compared to the NC + AAV8-EGFP group, the NC + AAV8-m-GPX4 group showed slight liver damage as well as hepatic lymphocyte infiltration in H&E-stained sections (Figure 3(e)). Also, ELISA analysis showed that the levels of the inflammatory cytokines TNF-*α*, IFN*γ*, and IL-17, and the fibrotic cytokine TGF-*β* were increased to some extent in the livers of the AIH + AAV8-EGFP group mice compared with the NC + AAV8-EGFP group, while the level of the anti-inflammatory cytokine IL-10 was decreased. After GPX4 knockdown, the expression of the inflammatory cytokines TNF-*α*, IFN*γ*, and IL-17 and the fibrotic cytokine TGF-*β* were upregulated to a greater extent in the livers of the AIH + AAV8-m-GPX4 group than those of the AIH + AAV8-EGFP group, while the anti-inflammatory cytokine IL-10 level was significantly downregulated (Figure 3(f); *P* < 0.05). In addition, Western blotting revealed that, in comparison to the NC + AAV8-EGFP group, the protein expression of COX2 and ACSL4 were strongly increased in the AIH + AAV8-EGFP group, while the protein expression of GPX4 and FTH1 were downregulated. The increase in COX2 and ACSL4 levels was greater in the AIH + AAV8-m-GPX4 group compared with the AIH + AAV8-EGFP group after GPX4 knockdown, while the relative protein expression of FTH1 was also significantly reduced (Figures 3(g) and 3(h); *P* < 0.05). The immunohistochemical results showed that COX2 staining in hepatocytes was significantly enhanced in the AIH + AAV8-m-GPX4 group compared to the AIH + AAV8-EGFP group, while GPX4 staining in hepatocytes was strongly reduced in the former in comparison to the AIH + AAV8-EGFP group. In addition, COX2 staining in hepatocytes was slightly enhanced in the NC + AAV8-m-GPX4 group compared to the NC + AAV8-EGFP group, while GPX4 staining in hepatocytes was reduced compared to the NC + AAV8-EGFP group in the former (Figures 3(i) and 3(j); *P* < 0.05). In terms of the lipid peroxidation malondialdehyde assays, MDA expression was increased to some extent after GPX4 knockdown in both AIH model groups with and without S100 induction (Figure 3(k); *P* < 0.05). Also, GPX4 knockdown increased the levels of ferrous ions to some extent (Figure 3(l); *P* < 0.05). This suggests that ferroptosis in S100-induced autoimmune hepatitis may be regulated through GPX4.

### 3.5. LPS-Induced Ferroptosis in AML12 Cells Occurs through GPX4 Regulation

To further investigate the mechanism of hepatic ferroptosis, LPS induction in AML12 hepatocyte cells was analyzed.

**Figure 5:**
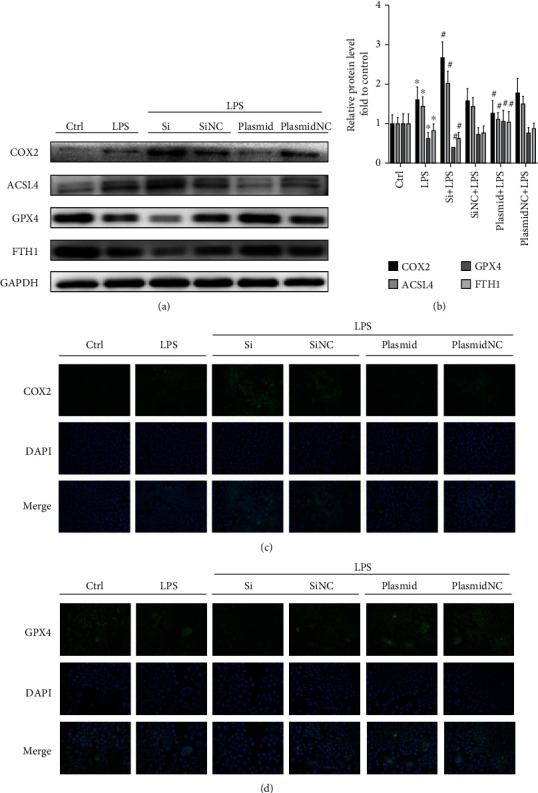
LPS-induced ferroptosis in AML12 cells occurs through the regulation of GPX4. (a) and (b) Western blot showing protein expression of COX2, ACSL4, GPX4, and FTH1 in the control, LPS, Si + LPS, SiNC+LPS, Plasmid+LPS, and PlasmidNC+LPS groups; GAPDH was used as a loading control; (c) and (d) COX2 and GPX4 immunofluorescence detection combined with DAPI staining for nuclei (scale bar: 75 *μ*m).^∗^*P* < 0.05, compared with the control group; ^#^*P* < 0.05, compared with the LPS group.

The experimental groups investigated were as follows: (i) control group, (ii) LPS-induced hepatocyte ferroptosis model group, (iii) LPS-induced hepatocyte ferroptosis group after GPX4-specific knockdown, (iv) LPS-induced hepatocyte ferroptosis group after nonspecific control siRNA transfection, (v) LPS-induced hepatocyte ferroptosis group after GPX4-specific plasmid overexpression, and (vi) LPS-induced hepatocyte ferroptosis group after nonspecific control plasmid transfection. Western blot analysis showed that the protein levels of COX2 and ACSL4 in the LPS-induced hepatocyte ferroptosis group were significantly raised, in contrast to those of GPX4 and FTH1 (Figures 5(a) and 5(b); *P* < 0.05). Furthermore, it is noteworthy that the protein levels of COX2 and ACSL4 increased after GPX4-specific knockdown siRNA transfection followed by LPS-induced hepatocyte ferroptosis compared to the LPS-induced hepatocyte ferroptosis model group alone, with a greater increase in comparison to the controls. Levels of FTH1 were also downregulated to a greater extent after GPX4 knockdown. Notably, after transfection with GPX4-specific overexpression plasmids followed by the same concentration of LPS to induce hepatocyte ferroptosis, COX2 and ACSL4 expressions were downregulated in comparison to the LPS-induced hepatocyte ferroptosis model group alone, and more so when compared to the control, while the levels of FTH1 also increased with GPX4 overexpression.

Cellular immunofluorescence staining showed that COX2 staining (green) was abundant in the LPS-induced hepatocyte ferroptosis model group, mainly localized in the cytoplasm. In addition, after GPX4 knockdown followed by LPS-induced hepatocyte ferroptosis, there was more intense COX2 staining compared to the LPS-induced hepatocyte ferroptosis model group alone, while COX2 staining after GPX4 overexpression followed by the same concentration of LPS-induced hepatocyte ferroptosis was significantly diminished compared to the LPS-induced hepatocyte ferroptosis model group alone (Figures 5(c) and 5(d)). These results suggest that GPX4 regulates the induction of LPS-induced ferroptosis in AML12 cells.

## 4. Discussion

The liver is the main iron storage site in the body, with iron in the form of ferritin and iron-containing hemoglobin. In the healthy liver, ferritin forms the main form of storage, with minimal availability of iron-containing heme. However, in the iron-overloaded liver, there are large accumulations of both ferritin and heme, especially in the case of hereditary or secondary diseases [21]. The evidence suggests that ferritin's role is to capture “free” iron within its spacious storage core, thus, protecting the cell against potential damage caused by reactive oxygen radicals generated by the Fenton reaction [22]. Several recent studies have shown that oxidative stress induced by ROS is the underlying pathophysiological mechanism in several liver diseases [23]. High levels of ROS lead to DNA damage, protein denaturation, and lipid peroxidation of and cyclooxygenase (COX) and lipoxygenase (LOX), together with affecting other enzyme functions [24]. Ferroptosis, a newly discovered type of iron-dependent nonapoptotic cell death, is biologically and morphologically distinct from apoptosis, necroptosis, and autophagy [25]. It is also characterized by free ferrous iron overload and the accumulation of lipid peroxides. However, lipid peroxide accumulation is mainly caused by the absence, or insufficient activity, of the selenium peroxidase glutathione peroxidase 4 (GPX4). GPX4 is a unique intracellular antioxidant enzyme that inhibits the production of lipid peroxidation in the cell membrane [26]. Given that the dynamic balance of iron is intimately linked to the maintenance of human health, disturbances in iron homeostasis can easily lead to a variety of medical conditions [27]. This investigation focused on the influence of ferroptosis in AIH and its possible regulatory mechanisms in the disease.

An in vivo mouse AIH model was established using S100, with the treated mice showing elevated serum ALT and AST levels, suggesting hepatic impairment. Elevation of serum IgG after 28 days indicated the presence of AIH. Meanwhile, the levels of the inflammatory cytokines TNF-*α*, IFN*γ*, and IL-17, and the fibrotic cytokine TGF-*β*, which play important roles in the pathology and progression of AIH, were increased, while the anti-inflammatory cytokine IL-10 level was decreased, further suggesting the presence of liver inflammation and fibrosis in the S100-induced AIH experimental mice. Specific inflammatory cytokines induce the activity of specific transcription factors that direct the differentiation of the relevant immune cell subtypes (Th1 and Treg cells secrete IFN*γ* and TGF-*β*, respectively, while Th17 cells secrete IL-17) [28]. There is growing evidence that both IL-17 and Th17 cells play key roles in the development and progression of AIH inflammation and that Th17 cells are important for the body's defense response [29]. Whereas the cellular source of TNF-*α* has not been specifically elucidated, several studies have also been reported suggesting that TNF-*α* may be released by activated monocytes, T cells, NK cells, mast cells, B cells, and Kupffer cells in the liver [30, 31]. In addition, histological analysis demonstrated inflammatory infiltration in the liver after S100 intervention that was consistent with a significant increase in COX2 expression. Genome-wide CRISPR-based genetic screening and microarray analysis of ferroptosis-resistant cell lines has previously asserted that ACSL4 is a key molecular player in the ferroptotic process [12]. Furthermore, stabilized glutathione levels in normal cells provide protection against oxidative stress and ferroptosis-driven cellular damage. Lipid peroxidation may increase as a consequence of either a reduced GPX4 expression or a reduction in the level of its cofactor glutathione [32]. At the onset of ferroptosis, increased iron-dependent lipid ROS production overwhelm GPX4's ability to control polyunsaturated fatty acid peroxidation, resulting in aberrant control of lipid peroxides and hence peroxidation, which are hallmarks of ferroptosis and lead to cell death [33]. It has been demonstrated in several studies that GPX4 is a major target of ferroptosis. There is direct genetic evidence that GPX4 knockouts lead to cell death in a pathologically relevant form of ferroptosis [34]. Recently, it was shown that cell death by ferroptosis is triggered by the knockout of GPX4 in either kidney or T cells [35]. Coincidentally, previous studies have shown that GPX4 overexpression and knockdown modulated the lethality of multiple ferroptosis inducers, but not of compounds associated with other cell death mechanisms [36]. Knockdown of FTH1 in hepatocellular carcinoma cells increased the incidence of ferroptosis, suggesting that FTH1 may play an important protective role in cellular ferroptosis and that reduced homeostasis in iron stores during the onset of ferroptosis may lead to iron overload [37]. Thus, aberrant expression of FTH1 may lead to issues associated with iron storage and cell death through the disruption of antioxidant defense systems [38]. The S100-induced AIH model group, in this investigation, showed upregulation of both COX2 and ACSL4, together with downregulation of GPX4 and FTH1. Ferroptosis is known to be activated through iron-dependent lipid peroxidation [39]. Therefore, we quantified the level of lipid peroxidation in the liver using malondialdehyde (MDA). The dysregulation of COX2, ACSL4, GPX4, and FTH1 expression, as well as MDA and iron overload levels, suggest an important role for ferroptosis in S100-induced autoimmune hepatitis.

To verify the role of ferroptosis, we used the ferroptosis inhibitor Ferrostatin-1 for therapeutic intervention in the S100-induced AIH mice. It was found that Ferrostatin-1 significantly attenuated the ALT, AST, and IgG serum levels, as well as the level of microscopic inflammatory infiltrates in H&E-stained sections in the S100-induced AIH mice. In addition, the expression of ferroptosis biomarkers (including ACSL4, GPX4, and FTH1) was significantly reduced by Ferrostatiin-1 treatment. Furthermore, iron-dependent oxidative stress and lipid peroxidation are common features of ferroptosis and inflammatory diseases [40]. Ferrostatin-1 reduced MDA levels in the AIH mouse livers. Thus, our data clearly illustrate that ferroptosis may be the main underlying mechanism that mediates S100-induced autoimmune hepatitis.

Nrf2, a master controller of the antioxidant response, is a transcription factor that is often aberrantly regulated in oxidative stress [41]. The stability of inactivated Kelch-like ECH-associated protein 1 (Keap1)/Nrf2 heterodimers is maintained by thiol antioxidants in the cytosol under nonstress conditions [42]. However, during oxidative stress, Nrf2 is dissociated from the Keap1 heterodimer in the cytosol, allowing Nrf2 to translocate to the nucleus where Nrf2 interacts with antioxidant response elements (ARE) to trigger the transcription of target genes (including HO-1) to mitigate oxidative stress [43]. In the present study, S100 induced an increase in hepatic Nrf2/HO-1 expression in the mouse AIH models. Furthermore, treatment with ferrostatin-1 reversed these effects, indicating that ferrostatin-1 may attenuate S100-induced AIH through inhibition of Nrf2/HO-1 pathway-mediated ferroptosis. These data suggest that the Nrf2/HO-1 pathway plays a pivotal role in thwarting ferroptosis. This highlights the role of ferroptosis in S100-induced AIH, with ferrostatin-1 ameliorating the AIH condition, mediated by the Nrf2/HO-1 signaling pathway.

Our investigation delved deeper into possible mechanisms of ferroptosis in AIH. The regulation of GPX4 in in vitro cellular experiments and in vivo animal experiments was assessed. AAV8-m-GPX4 was administered to S100-induced AIH mice to suppress GPX4 expression by liver-specific knockdown of the GPX4 gene. Tail-vein injection of AAV8-m-GPX4 to silence GPX4 expression resulted in increased levels of serum ALT, AST, and IgG, as well as increased infiltration of inflammatory cells. In addition, the expression of ferroptosis biomarkers (including COX2, ACSL4, and FTH1) was significantly increased in mice with S100-induced autoimmune hepatitis after specific knockdown of GPX4. Previous studies have shown that localized GPX4 deficiency can lead to increased levels of lipid peroxidation [44]. In the current study, liver-specific GPX4 knockdown also increased the MDA and iron-overload levels in the livers of S100-induced AIH mice. It has recently been shown that cell death due to ferroptosis is caused by a deletion of the GPX4 gene in the kidney [35]. The data presented in our study clearly demonstrate that GPX4 is vital for preventing the deleterious effects of lipid peroxidation and ferroptosis in autoimmune hepatitis. The in vitro cell experiments used GPX4-specific knockdown siRNA and a GPX4-specific overexpression plasmid to regulate LPS-induced ferroptosis model in AML12 cells. It was found that the expression of other ferroptosis biomarkers (including COX2, ACSL4, and FTH1) were significantly enhanced after GPX4 knockdown. Consequently, these results of this investigation implicate ferroptosis as an initiator or mediator of AIH pathogenesis, and that the occurrence of ferroptosis in AIH is regulated by GPX4.

## 5. Conclusions

In conclusion, our results demonstrate an essential role for ferroptosis in S100-induced AIH pathogenesis. It was also demonstrated that the Nrf2/HO-1 signaling pathway play a key part in inhibiting ferroptosis. Notably, S100-induced ferroptosis in AIH was found to be closely linked to GPX4 regulatory control. These results increase our knowledge of the molecular interactions underlying AIH and suggest directions for the development of novel therapeutic strategies to treat the disease.

## Data Availability

The data will be available upon reasonable request.
